# Does multiple gastric aspirate collection increase sensitivity of *M. tuberculosis* detection in children with pulmonary tuberculosis?

**DOI:** 10.1007/s00431-023-05277-6

**Published:** 2023-11-04

**Authors:** Elisabetta Venturini, Barbara Bortone, Gianmaria Cini, Jacopo Venanzi, Roberta Pellegrino, Anna Maria Bartolesi, Guendalina Vaggelli, Sandra Trapani, Giuseppe Indolfi, Leila Bianchi, Carlotta Montagnani, Elena Chiappini, Gian Maria Rossolini, Luisa Galli

**Affiliations:** 1 Infectious Diseases Unit, Meyer Children’s Hospital IRCCS, Florence, Italy; 2 Microbiology and Virology Unit, Careggi University Hospital, Florence, Italy; 3 Department of Health Sciences, University of Florence, Meyer Children’s Hospital IRCCS, Viale Pieraccini, 24 – 50139 , Firenze, Florence, Italy; 4 Neurofarba Department, University of Florence, Florence, Italy; 5 Department of Experimental and Clinical Medicine, University of Florence, Florence, Italy

**Keywords:** Gastric lavage, Children, Tuberculosis, Sensitivity

## Abstract

This study aims to investigate the sensitivity of microscopy, culture and polymerase chain reaction on three gastric aspirates (GAs) in the microbiological confirmation of active pulmonary tuberculosis (TB) and to identify possible changes in sensitivity derived from the collection of a different number of aspirates. Children with clinical and radiological diagnoses of active pulmonary TB who underwent three GAs between March 2007 and June 2019 were retrospectively evaluated. Clinical, radiological, and microbiological data were collected. The sensitivity of microbiological tests on GAs was calculated. Moreover, differences in sensitivity according to age and radiological pattern were investigated. Overall, 156 children with active pulmonary TB were enrolled with a median age of 51.5 (IQR: 25.2–113.2) months. Microbiological investigations on the first GA showed a sensitivity of 34% (95%CI 26.7, 42), the cumulative sensitivity of first and second GAs was 40.4% (95%CI 32.7, 48.5) and of the three GAs was 47.4% (95%CI 39.8, 55.2). The collection of three GAs leads to an overall increase in sensitivity of the first GA by 13.4% (95%CI 2.8, 24.1%; p=0.014). Moreover, the increase in sensitivity was significantly higher in children ≤ 4 years of age and in those with uncomplicated TB (p=0.008).

*Conclusions*: Performing a higher number of GAs increases the sensitivity of microbiological confirmation of active pulmonary TB, particularly in children ≤ 4 years and with an uncomplicated radiological pattern.

**Table:** 

**What is known:** *• The diagnosis of paediatric tuberculosis is a challenge for paediatricians* *• Despite their low sensitivity gastric aspirates represent the standard sample for microbiological confirmation of active pulmonary tuberculosis in children* *• Most international guidelines recommend performing three sequential gastric aspirates on three consecutive days*	
**What is new:** *• A significant increase in global sensitivity by 13.4% was found by the collection of three gastric aspirates compared to the first one* *• Performing a higher number of gastric aspirates increases the sensitivity of microbiological confirmation, particularly in children ≤ 4 years and with an uncomplicated radiological pattern*

**What is known:**

*• The diagnosis of paediatric tuberculosis is a challenge for paediatricians*

*• Despite their low sensitivity gastric aspirates represent the standard sample for microbiological confirmation of active pulmonary tuberculosis in children*

*• Most international guidelines recommend performing three sequential gastric aspirates on three consecutive days*

**What is new:**

*• A significant increase in global sensitivity by 13.4% was found by the collection of three gastric aspirates compared to the first one*

*• Performing a higher number of gastric aspirates increases the sensitivity of microbiological confirmation, particularly in children ≤ 4 years and with an uncomplicated radiological pattern*

## Introduction

Tuberculosis (TB) is a leading cause of morbidity and mortality worldwide, with over 440,000 new cases estimated in 2021 in children younger than 14 years [1]. Due to the COVID-19 pandemic a big drop in TB cases notification has been registered in the latter years. However, these data represent an underestimation of the real problem because of the issues posed by the diagnosis of TB in children. The detection of *Mycobacterium tuberculosis* in respiratory samples or other biological specimens confirms the diagnosis of TB and provides relevant information regarding the risk of transmission and antibiotic susceptibility [2, 3]. Pulmonary TB in children is often paucibacillary, making the diagnosis even more challenging. Moreover, young children are not able to produce sputum because of poor strength and motor coordination [4]. For these reasons, gastric aspirate (GA) represents the standard sample for microbiological confirmation of active pulmonary TB in children who cannot produce spontaneous or induced sputum (IS) [2].

The conventional microbiological tests performed on GAs are the cultural and the microscopic examination [3, 5, 6]. Although the collection technique has been standardized, the sensitivity of GAs in microbiological confirmation of pulmonary TB is globally less than 40% [4–7]. In order to increase the sensitivity, most international guidelines recommend performing three sequential GAs on three consecutive days [2, 3, 6, 8]. However, collecting GA can be invasive and distressing for children and may require hospitalization. In the last decades, molecular detection of *Mycobacterium tuberculosis* through Polymerase Chain Reaction (PCR) has been introduced and lead to a gain in diagnostic sensitivity [8–10].

This study aimed to investigate the sensitivity of the combination of microscopy, culture and PCR on three GAs in the microbiological confirmation of active pulmonary TB, in a cohort of children, from a low-prevalence setting, admitted to a tertiary care paediatric hospital.

## Materials and methods

### Study design and population

In this retrospective study, all children (0-18 years) with the diagnosis of active pulmonary TB -as defined below-, who underwent three GAs, because they did not have spontaneous expectoration, between March 2007 and June 2019 at the study centre were enrolled. Patients who underwent GAs that were finally judged to not have TB were excluded. According to the local ethical board all parents had signed the informed consent for children’s data inclusion in observational studies with anonymised data extraction. Demographic, clinical, radiological and microbiological data were collected from medical charts. The result of microscopy, culture and PCR on each GA was recorded. Moreover, the results of IFN-γ releasing assays (IGRA), tuberculin skin test (TST), chest radiograph (CXR), and chest tomography (CT) closest in time to the first GA were registered. All these records were entered into the study database following international standards for data protection.

### Case definition

A TST was performed in all children, by trained nurses, injecting 5 UI of PPD-S through the Mantoux method. After 48-72 hours, a pediatrician assessed the result. The TST was defined positive in the case of an induration ≥5 mm in children suspected to have TB disease, immunosuppressed or with a close TB contact, ≥10 mm in children <4 years of age, coming/travelling from high TB burden countries, with underlying chronic conditions, and ≥15 mm in children without any risk factors [2]. The IGRAs performed to assess the diagnosis were the QuantiFERON-TB Gold In-Tube from 2007 to 2015 and the QuantiFERON-TB Gold Plus from 2015.

Pulmonary TB was diagnosed according to the American Academy of Pediatrics criteria, i.e: close contact with a confirmed TB case and/or positive IGRA/TST and/or suggestive signs/symptoms and radiological findings of pulmonary TB [2]. The following radiological findings were considered suggestive of TB in children and adolescents: hilar lymphadenopathy, broncopneumonic consolidation, pleural effusion, cavitation, miliary disease, atelectasis and intrabronchial involvement. Active pulmonary TB was defined as “microbiologically confirmed” if at least one microbiological test (microscopy, culture, PCR) was positive in at least one respiratory sample. Otherwise, it was defined as “not microbiologically confirmed” [11, 12].

Findings from CXR and, if available, CT were defined as “complicated TB” according to the radiological classification proposed by Marais et al. [13]. Moreover, for the present study purpose, we extended the definition of “complicated TB” to miliary disease. Therefore, patients with segmental collapse or hyperinflation, miliary disease, cavitation, or intra-bronchial spread with multiple consolidations, were considered “complicated TB” cases [13].

### Gastric aspirate

A standardized protocol for GAs was applied. The procedure was performed, by a trained nurse, on three consecutive days with the patient in supine position and on an empty stomach. At least 5-10 ml of gastric fluids were collectedusing a 8 French nasogastric tube. In the case of scarce gastric fluid, the sample was collected after instillating 10 ml of normal saline solution. The sample was refrigerated and sent to the laboratory within 4 hours and no buffering or pH adjustments were applied. For the purposes of the present study, we defined 1^st^ GA, 2^nd^ GA and 3^rd^ GA as the first, the second, and the third GA performed, respectively.

### Microbiological tests

A direct smear fluorescence microscopy was performed, after auramine-rhodamine staining of the samples, Lowenstein-Jensen medium and *Mycobacteria Growth Indicator Tube* (MGIT) (Becton, Dickinson, and Company) were used for cultures, and samples were incubated for 60 or 42 days, respectively. *Mycobacterium* species were identified using Line Probe Assay (Hain), and antibiotic susceptibility was assessed through MGIT. PCR was performed to detect the *Mycobacterium tuberculosis* complex genome in GAs. During the study period, several assays for molecular detection were used (*Cobas-Roche, Artus-Qiagen, Gene Xpert MTB/RIF* and *Gene Xpert MTB/RIF Ultra- Cepheid*).

### Statistical analyses

Quantitative data were reported as absolute numbers and percentages or median and interquartile range (IQR), as appropriate. Differences among groups were calculated using the χ2 or Fisher’s test for categorical variables and the *Mann–Whitney* test for continuous variables. A *p* value <0.05 was considered significant.

The sensitivity of each microbiological test was calculated for each GA with the correspondent confidence intervals (95% CI). Furthermore, the cumulative sensitivity of microbiological detection was calculated for each GA, for the first two (GA 1-2), and for all the three GAs (GA 1-3). Sensitivity was calculated in relation to children diagnosed with tuberculosis disease according to the abovementioned criteria.

Univariable and multivariable logistic regression analyses were used to identify the variables influencing the sensitivity of microbiological detection on GAs, and risk was expressed as Odds Ratio (OR) with 95% CI.

All statistical analyses were carried out using SPSS package (Statistical Package of Social Science, Chicago, IL), version 27.0.

## Results

### Patients characteristics

**Fig.1:**
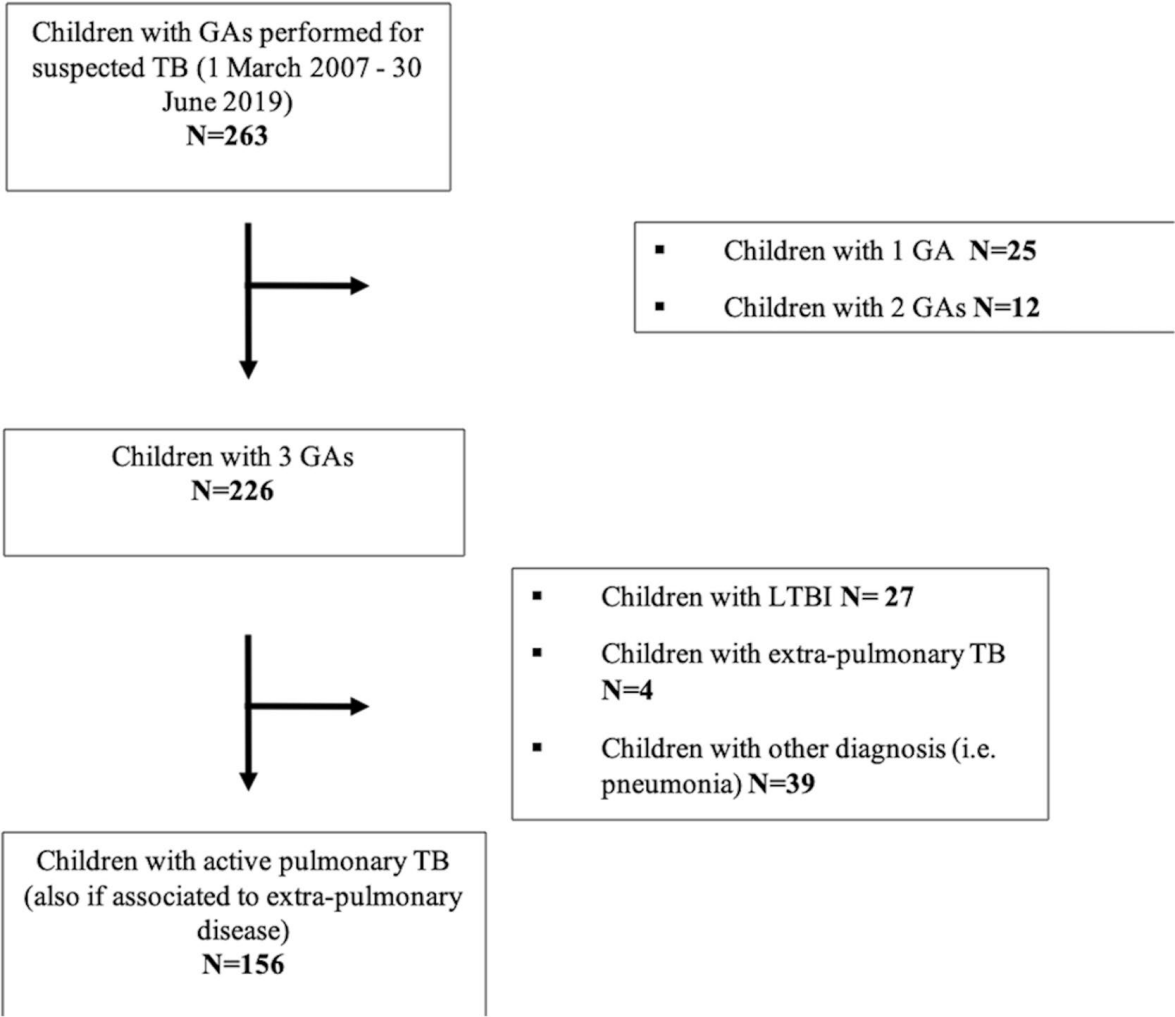
*Flow chart* of cases selection

Overall, 156 children with active pulmonary TB were enrolled. Of those, 46.8% were males, and the median age was 51.5 months (IQR: 25.2–113.2). Figure 1 shows the flow chart of cases selection*.

Among 156 enrolled patients, 143 (92%) were diagnosed with isolated pulmonary TB, whereas 13 patients had both pulmonary and extra-pulmonary TB. All patients were HIVnegative. The demographic and clinical characteristics of the study participants are shown in Table 1. Seventy-four (47.4%) patients had microbiologically confirmed pulmonary TB; the median age of the group was 60.9 months (IQR: 20.7-153.2). , Overall, children with negative GAs (52.6%) were younger, with a median age of 47.4 months (IQR: 25.5-107.4) (p=0.823). However, considering the microbiological confirmation among the different age groups, it was more frequent among children below 1 year (p=0.026) compared to the other groups.

**Table 1:** Demographic and clinical characteristics of the study participants.

	**All**	**Microbiologically confirmed**	**Not microbiologically confirmed**	**p**
	**n=156**	**n=74**	**n=82**	
**Sex**				
Male	73 (46.8%)	37 (50.7%)	36 (49.3%)	0.446
Female	83 (53.2%)	37 (44.6%)	46 (55.4%)	
**Age (months)**				
Median (IQR)	51.5 (25.2-113.2)	60.9 (20.7-153.2)	47.4 (25.5-107.4)	0.865
**Age distribution (years)**				
≤1	13 (8.3%)	10 (76.9%)	3 (23.1%)	**0.026**
2-4	62 (39.7%)	23 (37.1%)	39 (62.9%)	**0.036**
5-13	52 (33.3%)	23 (44.2%)	29 (55.8%)	0.57
>14	29 (18.6%)	18 (62.1%)	11 (37.9%)	0.08
**Country of origin**				
West Europe	26 (16.7%)	11 (42.3%)	15 (57.7%)	0.566
East Europe	37 (23.7%)	16 (43.2%)	21 (56.7%)	0.558
Africa	49 (31.4%)	17 (34.7%)	32 (65.3%)	**0.031**
Asia	21 (13.5%)	12 (57.1%)	9 (42.9%)	0.338
South-central America	23 (14.7%)	18 (78.3%)	5 (21.7%)	**0.001**
**Reason for investigation**				
Contact with confirmed TB patient	93 (59.6%)	43 (46.2%)	50 (53.8%)	0.715
Adoption/immigration screening	9 (5.8%)	1 (11.1%)	8 (88.9%)	**0.024**
Symptomatic	54 (34.6%)	30 (55.6%)	24 (44.4%)	0.139
**BCG vaccination**				
Yes	12 (7.7%)	3 (4.1%)	9 (11%)	**0.005**
No	78 (50%)	38 (51.3%)	40 (48.8%)	
Unknown	66 (42.3%)	33 (44.6%)	33 (40.2%)	
**TST**				
Positive	145 (92.9%)	68 (46.9%)	77 (53.1%)	0.686
Negative	11 (7.1%)	6 (54.5%)	5 (45.5%)	
**IGRA results**				
Positive	135 (86.5%)	66 (48.9%)	69 (51.1%)	0.245
Negative	20 (12.8%)	7 (35%)	13 (65%)	
Indeterminate	1 (0.6%)	1 (100%)	0 (0%)	

Bold values indicate statistical significance at the p < 0.05 level

The proportion of microbiologically confirmed GAs according to the radiological features is shown in Table 2. Overall, 80.1% (125/156) of the children showed an uncomplicated radiological picture. No statistically significant difference in microbiological confirmation was found in complicated *vs.* uncomplicated cases (p=0.09). Analysing all radiological patterns, parenchymal cavitation was more frequently associated with microbiologically confirmed cases when compared to negative ones (86.7% *vs.* 13.3%, p=0.001). All patients with miliary TB were microbiologically confirmed (p=0.022). Microbiological confirmation was more likely in children with parenchymal cavitation (p=0.001) and miliary disease (p=0.022). On the contrary, children with hilar lymphadenopathy were more likely to have microbiologically negative GAs (p=0.041), as shown in Table 2.

**Table 2:** Microbiological confirmation according to radiological features.

		**All** **(n=156)**	**Microbiologically confirmed** **(n=74)**	**Not microbiologically confirmed** **(n=82)**	**p**
**Uncomplicated (n=125)**	**Hilar lymphadenopathy**	22 (14.1%)	6 (27.3%)	16 (72.7%)	**0.041**
	**Bronchopneumonic consolidation**	23 (14.8%)	9 (39.1%)	14 (60.9%)	0.387
	**Bronchopneumonic consolidation** **+** **Hilar lymphadenopathy**	74 (47.4%)	37 (50%)	37 (50%)	0.542
	**Pleural effusion** **+** **Bronchopneumonic consolidation** **+** **Hilar lymphadenopathy**	6 (3.9%)	1 (16.7%)	5 (83.3%)	0.213
**Complicated (n=31)**	**Parenchymal cavitation**	15 (9.6%)	13 (86.7%)	2 (13.3%)	**0.001**
	**Miliary disease**	5 (3.2%)	5 (100%)	0 (0%)	**0.022**
	**Atelectasis**	8 (5.1%)	3 (37.5%)	5 (62.5%)	0.722
	**Intra-bronchial involvement**	3 (1.9%)	0 (0%)	3 (100%)	0.247

Bold values indicate statistical significance at the p < 0.05 level

### Performance of microbiological tests on GAs

Microbiological tests showed a sensitivity of 34% (95%CI 26.7, 42) for 1^st^GA, 32.1% (95%CI 24.9, 40.1) for 2^nd^GA and 35.9% (95%CI 28.5, 44) for 3^rd^GA (1^st^GA *vs.* 2^nd^GA p=0.722; 1^st^GA *vs.* 3^rd^GA p=0.725; 2^nd^GA *vs.* 3^rd^GA p=0.479). The cumulative sensitivity of GA (1-2) was 40.4% (95%CI 32.7, 48.5), while that of GA (1-3) was 47.4% (95%CI 39.8, 55.2).

**Fig.2:**
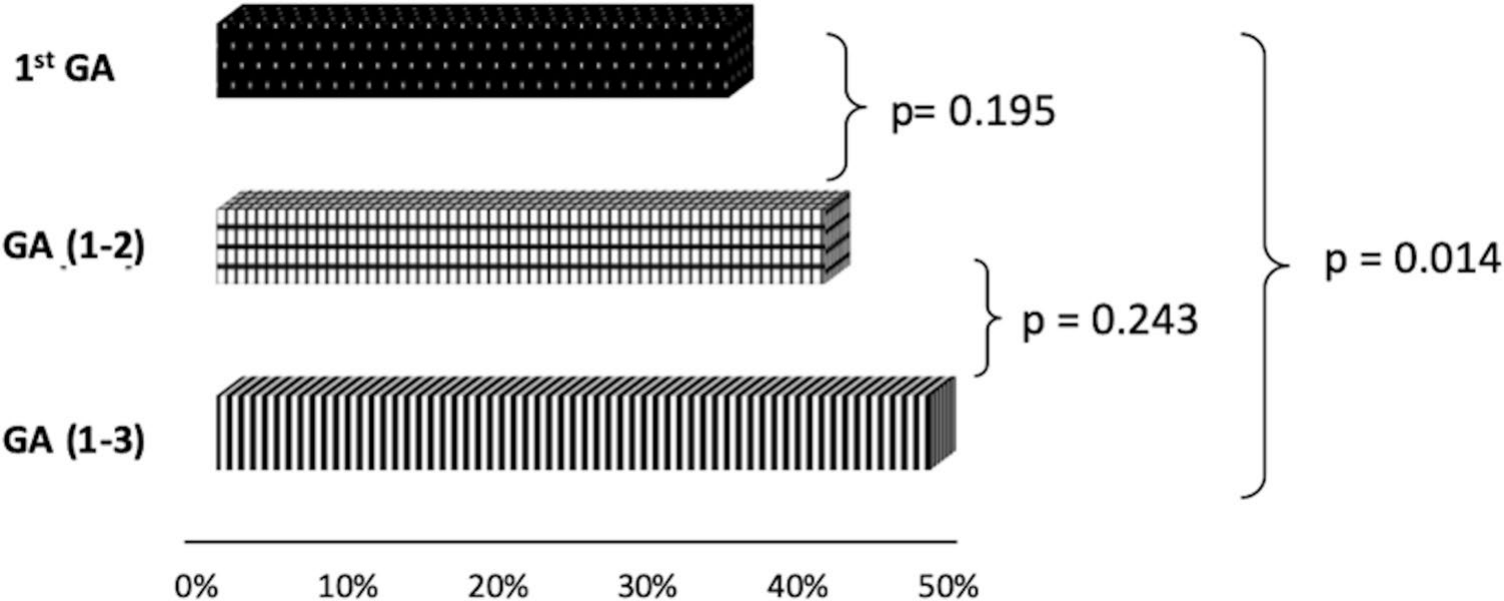
Sensitivity of 3 GAs in microbiological confirmation of active pulmonary TB cases

Therefore, the collection of the 2^nd^GA increased the sensitivity of the 1^st^GA by 6.4% (95%CI -4.3, 16.9) (p=0.243), and the 3^rd^GA led to a further increase in sensitivity by 7% (95%CI -3.9, 17.7) (p=0.195), obtaining a microbiological confirmation in other 10 and 11 patients, respectively. The collection of both the second and third GAs led to a significant increase in sensitivity of 13.4% (95%CI 2.8, 24.1; p=0.014) compared to the first GA only (Fig. 2).

The sensitivity of each microbiological investigation (microscopy, PCR, culture) for each GA is reported in Table 3. Microscopy and culture were obtained in all the included patients, whereas PCR was performed in 150/156 children (96.1%). No statistically significant difference was found when comparing the sensitivity of each microbiological investigation among each gastric GA (Table 3).

**Table 3:** Positivity rate of microbiological investigations for each GA

	**1**^**st**^ **GA** n [%; 95% CI]	**2**^**nd**^ **GA** n [%; 95% CI]	**3**^**rd**^ **GA** n [%; 95% CI]	**p** **1**^**st**^ ***vs.*** **2**^**nd**^ **GA**	**p** **2**^**nd**^ **v*s.*** **3**^**rd**^ **GA**	**p** **3**^**rd**^ ***vs.*** **1**^**st**^ **GA**
Microscopy (n=156)	9 [5.8%; 3.1, 10.6]	12 [7.7%; 4.4, 12.9]	15 [9.6%; 5.9, 15.3]	0.504	0.551	0.208
PCR (n=150)	22 [14.7%; 9.9, 21.2]	25 [16.7%; 11.5, 23.4]	24 [16%; 10.9, 22.7]	0.635	0.870	0.755
Culture (n=156)	48 [30.8%; 24.1, 38.4]	46 [29.5%; 22.9, 37.0]	52 [33.3%; 26.4, 41.0]	0.803	0.470	0.637

**Fig.3:**
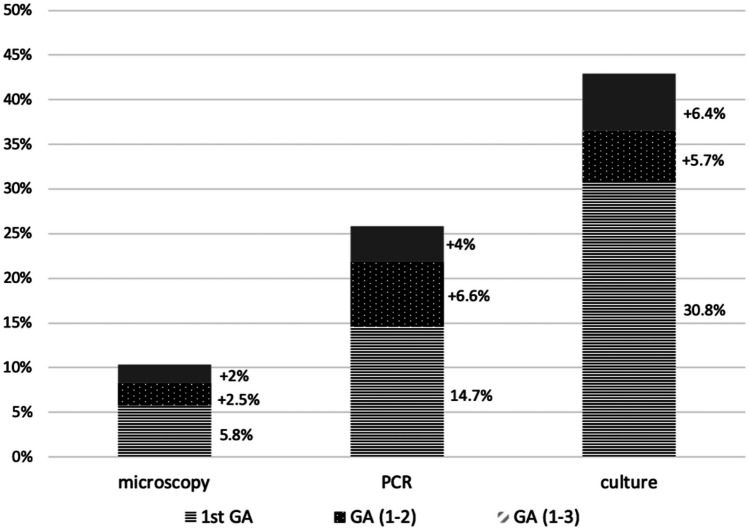
Sensitivity of each microbiologic test in relation to the number of gastric aspirates performed

The increase in sensitivity of each test related to the number of performed GAs is shown in Fig. 3. No significant difference in microscopy sensitivity was found between 1stGA vs. GA (1-2) (p=0.382), GA (1-2) vs. GA (1-3) (p=0.544), and 1stGA vs. all the three GAs (p=0.144). The sensitivity of PCR was significantly higher considering the three GAs compared to the first one (p=0.020), whereas no significant differences were found between 1stGA vs. GA (1-2) (p=0.634) and GA (1-2) vs. GA (1-3) (p=0.649).

The culture showed the highest sensitivity compared to the other tests, which increased significantly if performed on three GAs compared to the first one (p=0.027). On the contrary, the difference was not statistically significant when comparing 1^st^GA *vs*. GA (1-2) (p=0.297) and GA (1-2) *vs.* GA (1-3) (p=0.258).

**Fig.4:**
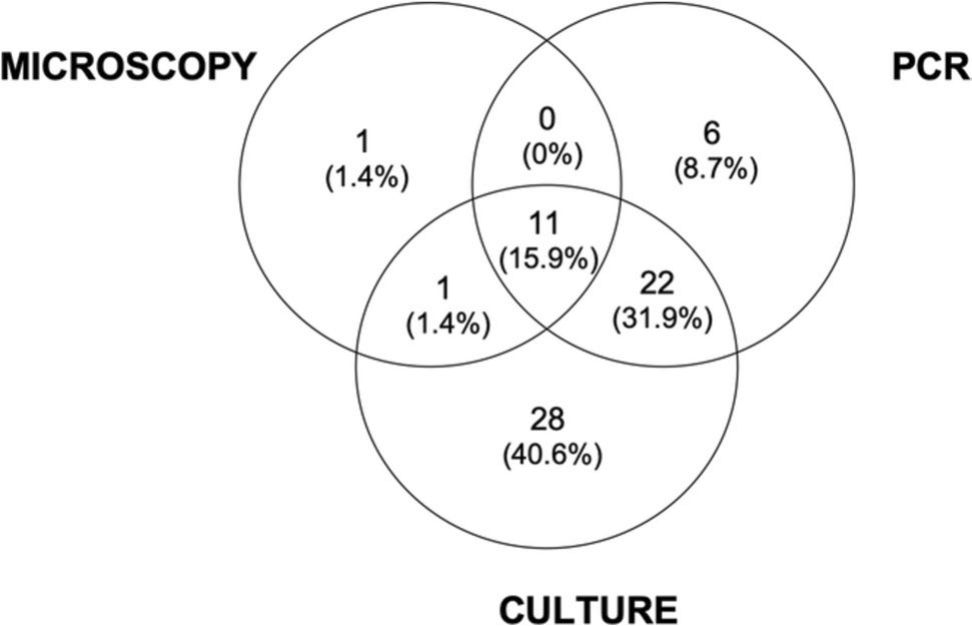
Positive results of each microbiological test, considering overall the three GAs

Positive results of each microbiological test, considering overall the three GAs, are shown in Fig. 4.

### Performance of GAs according to age groups and radiological classification

Sensitivity of GAs in children > and ≤ 4 years of age and with uncomplicated/complicated radiological pattern, according to the number of GAs performed, is reported in Table 4. Considering the age groups, in children younger than 4 years the microbiological confirmation obtained with GA(1-3) lead to a significant increase in sensitivity of 19.7% (95%CI 4.5, 33.6; p=0.0011) as compared to 1^st^GA, whereas there was not a significant difference in the proportion of microbiological confirmation on GA(1-2) *vs*. 1^st^GA (10.5%, 95%CI -4.1, 24.5; p=0.160) and GA(1-3) *vs*. GA(1-2) (9.2%, 95%CI -6.3, 24.1; p=0.248). In the group of children older than 4 years a significant increase in sensitivity was not observed in any case: GA(1-2) *vs*. 1^st^GA (2.5%, 95%CI -12.6, 17.4; p=0.751); GA(1-3) *vs*. 1^st^ GA (7.5%, 95%CI -7.8, 22.3; p=0.311); GA (1-3) *vs*. GA (1-2) (5%, 95%CI -10.3, 19.9; p=0.528). In children with uncomplicated TB, performing three GAs increased the sensitivity by 16% when compared to the 1^st^ GA (95%CI 4.2, 27.2; p=0.008). On the contrary, the increases in sensitivity in GA(1-2) *vs*. 1^st^ GA by an additional 7.2% (95%CI -4.1, 18.3; p=0.215), and between GA(1-3) *vs*. GA(1-2) by an additional 8.8% (95%CI -3.2, 20.4; p=0.152) were not statistically significant. In the multivariate analysis evaluating the role of the radiological classification and age on the overall microbiological performance, only the radiological classification resulted as an independent variable affecting the sensitivity of three GAs (radiological classification: OR 2.8, 95%CI 1.2, 6.5, p=0.013; age: OR 1.23; 95%CI 0.66, 2.32, p=0.511).

**Table 4:** Sensitivity of GAs according to age and radiological pattern

	**1**^**st**^** GA** % [95% CI]	**GA (1-2)** % [95% CI]	**GA (1-3)** % [95% CI]	**P (1**^**st**^** GA)**	**P (GA 1-2)**	**P (GA 1-3)**
**> 4 years**	42.5 32.3, 53.4	45 34.6, 55.9	50 39.3, 60.7	**p=0.021**	p=0.228	p=0.509
**< 4 years**	25 16.6, 35.8	35.5 25.7, 46.7	44.7 34.1, 55.9			
**uncomplicated TB**	26.4 19.5, 34.7	33.6 25.9, 42.3	42.4 34.2, 51.2	**p=0.0001**	**p=0.0006**	**p=0.014**
**complicated TB**	64.5 46.9, 78.9	67.7 50.1, 81.4	67.7 50.1, 81.4			

Bold values indicate statistical significance at the p < 0.05 level

## Discussion

This study evaluated the diagnostic yield of collecting three GAs for the microbiological confirmation of pulmonary TB disease in children from a low-prevalence and high-income setting. The sensitivity of each GA and the cumulative sensitivity was evaluated in relation to children diagnosed with pulmonary TB disease. The global sensitivity of the combination of microscopy, culture and PCR on three GAs in 156 children with active pulmonary TB was 47.4%. Interestingly, collecting three GAs significantly increased the sensitivity by 13.4% compared to the first GA alone. However, the second and third aspirates did not significantly increase the sensitivity individually.

The heterogeneity of the available studies, in terms of setting, number of collected samples and microbiological tests performed, only allows for a partial comparison to our data. Most studies carried out in high-prevalence and low-middle-income countries, [4, 13, 14] reported a heterogeneous and extremely wide range of sensitivity (1-45%), although molecular assays were rarely used [15]. Moreover, the diagnostic yield of each sample and the combination of different microbiological tests are rarely considered in the available studies [16–21]. In a previous retrospective study carried out in our centre on 102 children with pulmonary TB, the global sensitivity of the combination of the three GAs was 44.1% [16], in line with the present results. A retrospective German study including 454 children who underwent at least two GAs between 2002 and 2010, and performing both culture and PCR found a global sensitivity of 63%, higher than the sensitivity of 43.6% from our study. This difference might be related to a higher sensitivity of PCR (48% *vs.* 25.3%) [19]. Moreover, most of children in the German cohort were younger than 1 year of age (68% *vs*. 8% in our population). Infants are more likely than older children to develop a disseminated TB, this could explain the higher probability of microbiological confirmation [22]. Similarly, in our study, the microbiologically confirmed cases were frequently found among children younger than one year (76.9%) and those older than 13 years (62.1%), who could develop a cavitary disease as the adults.

In our population, the collection of three GAs led to a significant increase in sensitivity compared to one GA in the group of children ≤ 4 years and in patients with uncomplicated TB. However, this was not found in children older than 4 years and in those with complicated TB [13]. Since uncomplicated TB disease is often paucibacillary, collecting more GAs may increase the chance of microbiological confirmation.

In our study, a significant correlation between complicated radiological patterns, including miliary disease, and microbiological confirmation was found. On the contrary, children with isolated hilar lymphadenopathy were more likely to have negative microbiological results.

Kordy et al. previously investigated the association between radiological pattern and overall sensitivity of GAs, and reported that both miliary disease and hilar lymphadenopathy were predictive of positive GAs cultures [18]. However, the results by Kordy et al. are difficult to compare with our findings due to the different sample size and the sole use of culture as microbiological test [18].

A US retrospective study assessed the sensitivity of culture on each GAs [20]. In the latter study, among children with a positive culture on gastric aspirates, 24/32 (75%) grew *M. tuberculosis* from the first specimen obtained, with second and third GAs increasing diagnostic yield of 19% (6/32) and 8% (2/24), respectively [20]. A retrospective study, carried out in Canada from 1999 to 2011, included 202 children who underwent three GAs [18]. In the latter study, the cumulative culture-positive rate for those who had 3 GAs and were treated for TB disease was 31.7% as compared with 42.9% in our study. This difference might rely on the more stringent inclusion criteria used in our study. Of 15 culture-positive patients who underwent exactly 3 GAs, *M. tuberculosis* was isolated from the first sample in 10 (67%), only from the second in 3 patients (20%) and only from the third one in 2 (13%). A prospective Spanish pilot study on 17 children reported a global sensitivity of the combination of microscopy and culture of 47%, in line with our study [17]. Interestingly, the first GA had a higher sensitivity if compared with our results (41.2% *vs*. 34.1%), but this difference could not be clearly interpreted due to the small study population [17].

A systematic review of 30 studies, including 11,554 children, found that the sensitivity of culture and Xpert MTB/RIF on different respiratory samples were within the following ranges: 1-30% and 2-17% for IS, 1-45% and 5-51% for GA, and 4-24% and 3-8% for nasopharyngeal aspirate (NPA), respectively [15]. Collecting a second specimen contributed for 6%–33% of the cumulative yield and combination of different methods significantly increased the detection yields [15]. A recent meta-analysis on Gene Xpert MTB/RIF Ultra in children confirmed that sensitivity differs by specimen type, with sputum having the highest sensitivity, followed by gastric aspirate [21].

Samples other than GAs have also been evaluated, and stool is found to be a promising specimen [23, 24]. Hence, the World Health Organization recommends stool as an alternative to sputum or GA for Xpert MTB/RIF in adults and children with suspected pulmonary TB [23]. Spontaneous expectoration and induced sputum are usually obtained in adults for microbiological confirmation. Inducing sputum is considered a safe procedure also in children [25]. Nonetheless, children usually have immature motor coordination and lack of tussive force which could impair the sample collection. The diagnostic performance of IS versus GA is debated. However, combining different specimens could increase the diagnostic yield in young children, as shown in studies from lower-middle-income countries (LMICs) [26, 27]. A study assessing 300 Kenyan children showed that the combination of minimally invasive specimens (2 NPA, NPA plus stool sample, or NPA plus urine sample) led to a bacteriologic yield comparable to that of the reference-standard specimens (2 GAs or 2 ISs) [26]. On the other hand, specimen pooling has been assessed in a study including 304 children younger than 5 years in South Africa [27]. The overall diagnostic yield from pooled specimens (GA, NPA, and IS) was not different from that of a single GA, but it was significantly higher than a single IS or NPA specimen, suggesting that the GA was the main contributor to the diagnostic yield of pooled specimens [27]. These promising data regarding combined and pooled specimens should be confirmed in larger populations.

The main limitations of our study are its retrospective design and the limited number of enrolled children. The sample size is further reduced when stratifying the population based on age and radiological picture. This may explain some unexpected results, such as the fact that none of the children with intrabronchial involvement had microbiological confirmation. However, the highly selective inclusion criteria allowed the estimation of the sensitivity of the three microbiological tests on three GAs in a homogeneous group of children with pulmonary TB. Moreover, the application of a standardized protocol for GAs collection is likely to have limited the inter-operator variability of the procedure. During the study period, several molecular detection kits were employed. Variations in the sensitivity of these assays might have influenced the GA sensitivity. Due to the small sample size, it was not possible to obtain statistical information about the performance of GAs using different molecular assays. Nonetheless, every molecular assay utilized exhibited high sensitivity thereby mitigating potential impacts on our findings [21, 28, 29].

In conclusion, microscopy, culture, and PCR on three sequential GAs represent the gold standard for the microbiological confirmation of paediatric pulmonary TB in a low prevalence setting. The result of this study supports the practice of obtaining three GAs, and this continues to be our institutional practice. However, it is imperative to consider a child's medical history and clinical presentation, and the outcomes of other tests, notably IGRA, when dealing with microscopy-positive smears that are not confirmed by PCR or culture. This is necessary to discriminate between TB disease and NTM infection.

Performing a higher number of GAs might increase the sensitivity, particularly in children ≤ 4 years and in those with an uncomplicated radiological pattern. However, these results need to be confirmed by other studies with a prospective design and larger population. Similarly, larger studies on the diagnostic yield of different samples combinations are needed in order to find the best association able to reach the highest sensitivity with the less discomfort, especially for younger children.

## Data Availability

The dataset that support the findings of this study is available from the corresponding author upon reasonable request.

## Abbreviation

CT: Chest tomography
CNS: Central nervous system
CXR: Chest radiograph
GA: Gastric aspirate
IGRA: IFN-γ release assay
IS: Induced sputum
IQR: Interquartile range
MGIT: Mycobacteria Growth Indicator Tube
NPA: Nasopharyngeal aspirate
OR: Odds ratio
PCR: Polymerase chain reaction
TB: Tuberculosis
TST: Tuberculin skin test
